# The selective mGluR5 agonist CHPG protects against traumatic brain injury *in vitro* and *in vivo* via ERK and Akt pathway

**DOI:** 10.3892/ijmm.2011.870

**Published:** 2011-12-28

**Authors:** TAO CHEN, LEI ZHANG, YAN QU, KAI HUO, XIAOFAN JIANG, ZHOU FEI

**Affiliations:** 1 Department of Neurosurgery, Xijing Institute of Clinical Neuroscience, Xijing Hospital, Fourth Military Medical University, Xi'an, Shaanxi 710032; 2 Department of Surgery, The 123th Hospital of PLA, Bengbu, Anhui 233000, P.R. China

**Keywords:** apoptosis, neuroprotective effect, traumatic brain injury, extracellular signal-regulated kinase, Akt

## Abstract

Group I metabotropic glutamate receptors (mGluRs) have been implicated in the pathophysiology of central nervous system injury, but the role of mGluR5 in traumatic brain injury (TBI) remains unclear. In the present study, we investigated the neuroprotective potency of (R,S)-2-chloro-5-hydroxyphenylglycine (CHPG), a selective mGluR5 agonist, for protecting against TBI in both *in vitro* and *in vivo* models. Primary cortical neurons were treated with 1 mM CHPG in an *in vitro* preparation 30 min before TBI, and 250 nM CHPG was injected into the right lateral ventricle of rats 30 min before TBI was induced in *in vivo* studies. The results showed that CHPG significantly attenuated lactate dehydrogenase (LDH) release and neuronal apoptosis and reduced lesion volume. Compared to the control or vehicle group, the phosphorylation levels of extracellular signal-regulated kinase (ERK) and Akt were increased in the presence of CHPG, even following the induction of TBI. Furthermore, treatment with either the ERK inhibitor PD98059 or Akt inhibitor LY294002 partially reversed the CHPG's neuroprotective effects. These data suggest that CHPG minimizes brain damage after induction of TBI both *in vitro* and *in vivo*, and that these protective effects were possibly mediated by activation of the ERK and Akt signaling pathways. Thus, potentiating mGluR5 activity with selective agonists such as CHPG may be useful for the treatment of traumatic brain injury.

## Introduction

Traumatic brain injury (TBI) is one of the leading causes of death in the world and is considered to be a major cause of adult disability (1). It is reported that approximately 5 million new cases of TBI occur in the United States every year, with an estimated annual cost of $60 billion (2,3). After TBI, the primary mechanical injury leads to various pathological changes comprising the secondary brain injury, such as blood-brain barrier disruption, cerebral edema and increase of intracranial pressure, resulting in long-term and even permanent disability (4). The mechanisms leading to cell death after TBI are still not fully understood, and no proven pharmacological treatment exists (5,6).

Glutamate is an extensively distributed, primarily excitatory neurotransmitter in the mammalian nervous system. Excessively high extracellular glutamate concentrations, which are frequently found in the central nervous system (CNS) after TBI, appear to have an important role in secondary brain injury (7,8). The glutamate excitotoxicity is mediated by several glutamate receptor types, including ionotropic (ligand-gated cation channels) and metabotropic (G-protein coupled) receptors. The contribution of ionotropic glutamate receptors (iGluRs) to traumatic brain injury has been widely investigated (9), and several pharmacological antagonists of iGluRs show considerable neuroprotective effects in experimental investigations (10–12). Unfortunately, however, none of these candidate neuroprotective agents could translate the theoretical advantage into a real therapeutical benefit for TBI therapy in clinic, partly because these compounds also alter vital homeostatic functions that are modulated by the widely distributed iGluRs. With a more limited distribution and high concentrations in the CNS, the more recently discovered metabotropic glutamate receptors (mGluRs) may provide a better option to regulate excitatory neurotransmission without causing undesired side effects (13). The mGluRs are classified on the basis of amino acid sequence homologies, signal transduction pathways and pharmacological sensitivities into the following three groups: group I (mGluR1 and 5), group II (mGluR2 and 3) and group III (mGluR4, 6, 7, 8). Group I mGluRs are typically postsynaptically localized in somatodendritic domains and coupled to phosphoinositide (PI) hydrolysis and intracellular Ca^2+^ mobilization through phospholipase C (PLC). Several previous studies have demonstrated that antagonists of these receptors reduce neuronal damage after TBI (14–16), and the protective effects may be predominantly mediated by blockage of mGluR1 (17,18). In contrast to mGluR1, most studies of mGluR5 mainly focus on neurodegenerative diseases, and its role in neuronal cell death is controversial because of contradictory results obtained in different disease models (19–21). In the present study, a selective mGluR5 agonist (R,S)-2-chloro-5-hydroxyphenylglycine (CHPG), which does not activate mGluR1, was used to examine the specific contribution of mGluR5 to neuronal damage after TBI.

After traumatic brain injury, both pro-survival and pro-death pathways are triggered and the balance between these pathways determines the destination of injured cells and influences their functional recovery. A critical strategy for the treatment of TBI is to find compounds that can activate pro-survival signaling and inhibit pro-death mechanisms. On activation by phosphorylation, Akt and extracellular signal-regulated kinase (ERK), two well-characterized pro-survival molecules, are demonstrated to contribute to protective effects of many neuroprotectants (22,23). Furthermore, it has been suggested that mGluR can activate Akt and ERK in different models (24,25), but to the best of our knowledge no investigations of their relationship on TBI have been conducted. Thus, we examined the effects of CHPG on the activation of Akt and ERK, and by using specific inhibitors the potential mechanism of CHPG-induced neuroprotection against TBI was investigated in *in vitro* and *in vivo* models.

## Materials and methods

### Animals

Adult Sprague-Dawley male rats weighing 280–320 g were obtained from the Laboratory Animal Center of the Fourth Military Medical University. The animals had continuous access to food and water and were housed in cages in a room maintained at 20–22˚C with a 12 h light/12 h dark cycle. All experimental protocols and animal handling procedures were performed in accordance with the National Institutes of Health (NIH) guidelines for the use of experimental animals and approved by the Institutional Animal Care and Use Committee of the Fourth Military Medical University.

### Drug treatments

PD98059 and LY294002 (Cell Signaling Technology, Ozyme, France) were dissolved in DMSO and diluted in saline (1% final DMSO concentration). CHPG (Sigma, Saint Louis, MO, USA) was dissolved in saline. For the *in vitro* experiments, CHPG (1 mM), PD98059 (10 μM) or LY294002 (50 μM) was directly added into the culture medium 30 min before traumatic injury was induced. For the *in vivo* experiments, vehicle (1% DMSO in saline), CHPG (250 nM), PD98059 (5 nM) or LY294002 (15 nM) was injected in a volume of 5 μl into right lateral ventricle (anteroposterior, 0.8 mm; lateral, 1.5 mm; depth, 3.5 mm from bregma) 30 min before TBI.

### Primary cultures of cortical neurons

Cortical neurons were cultured from Sprague-Dawley rats using a modified method that has been previously described (26). Briefly, cortical tissue was removed from embryos at 16–18 days, and maintained in PBS at 4˚C during dissection. Tissues were dissociated by 0.25% trypsin digestion for 15 min at 37˚C and gentle trituration. Neurons were resuspended and plated onto poly-D-Lysine-coated (50 μg/ml) 60 mm culture dishes at a density of 3×10^5^ cells/cm^2^. The neurons were cultured in neurobasal medium (Gibco, Gaithersburg, MD, USA) containing 2% B27, 0.5 mM L-glutamine and 100 U/ml penicillin at 37˚C in a humidified 5% CO_2_ incubator and half of the culture medium was changed every other day. Cultures were utilized for experiments at 8–10 days when more than 95% of cells were cortical neurons as determined by immunofluorescence staining of neurofilament 200 (data not shown).

### In vitro trauma model

Our *in vitro* trauma model was based somewhat on the mechanical injury model described previously (17,27). Briefly, each 60 mm dish confluent culture was manually scratched with a sterile plastic pipette tip following a 20×20-square grid (with 3 mm spacing between the lines). These cuts caused immediate death to cells directly under the blades, followed by progressive secondary injury of neurons at a distance from these cuts. After injury, the cultures were washed with PBS to remove cellular debris, and then incubated for further 24 h at 37˚C in a humidified 5% CO_2_ incubator.

### Lactate dehydrogenase (LDH) measurement

The release of LDH, a cytoplasmic enzyme released from neurons with ruined cell membranes, was used as a marker of neuronal damage and was assessed 24 h after traumatic injury. The amount of LDH released into the medium was measured using a diagnostic kit according to the manufacturer's instructions (Jiancheng Bioengineering Institute, Nanjing, China). Pyruvate and reduced form of nicotinamide-adenine dinucleotide (NADH) were added into the medium samples from each group, and after 15 min of incubation at 37˚C, the reaction was stopped by adding 0.4 mol/l NaOH. The absorbance of the sample was read at 490 nm, and the results were expressed as a percentage of LDH release from the sample vs. the maximal value, which was determined by treating control cultures with 1% Triton X-100 for 60 min to lyse all cells.

### Identification of apoptotic neurons

Neuronal apoptosis was analyzed by staining the nuclear chromatin with Hoechst 33342 (Molecular Probes, USA). In brief, 24 h after TBI the culture medium was removed and neurons were washed with PBS. Hoechst 33342 (5 μg/ml) was added, and then neurons were maintained for 15 min at 37˚C in a CO_2_ incubator. Finally, these labeled neurons were observed using a Leica fluorescence microscope (B-251, Berlin, Germany), and the number of apoptotic cells with nuclear condensation and fragmentation were counted. Apoptotic rate is presented as the percentage of the total number of neurons.

### Traumatic brain injury in vivo

Traumatic brain injury *in vivo* was induced as previously described (28). In brief, rats were anesthetized using 2% isoflurane in oxygen and placed in the stereotaxic frame. A craniotomy was performed using a portable drill and a 5 mm trephine over the right parietotemporal cortex. The resulting bone flap was removed and the dura remained intact. To induce injury, a pneumatic piston impactor device with a 5 mm diameter and rounded tip (Biomedical Engineering Facility, Virginia Commonwealth University, Richmond, VA) was used to impact the brain at a depth of 2 mm for 250 ms. After injury, the bone flap was replaced and sealed with bone wax. Sham animals underwent similar anesthetic and surgical interventions, including craniotomy, but did not receive the TBI application. Core body temperature was continuously monitored with a rectal probe and maintained at 37˚C with a thermostatically controlled heating pad during surgery.

### TUNEL staining

Apoptosis in brain sections was detected by the TUNEL assay, a method used to observe DNA strand breaks in nuclei. In brief, after being washed in Tris-HCl (pH 7.7) three times, sections were treated with proteinase K solution (20 μg/ml) for 10 min at room temperature to permeabilize tissues. Sections were then labelled with fluorescein TUNEL reagent mixture for 60 min at 37˚C according to the manufacturer's suggested protocol (Promega Corporation, Madison, USA). The reactions were terminated by immersing the sections in 2X SSC buffer (0.3 M NaCl, 30 mM Na_3_C_6_H_5_O_7_, pH 7.0) for 15 min at room temperature. After that, sections were examined by fluorescence microscopy and the number of TUNEL-positive (apoptotic) cells was counted in five fields in each section.

### Lesion volume assay

Lesion volume was measured 7 days after TBI. Rats were anesthetized with 4% isoflurane in oxygen and decapitated. The brains were rapidly removed and cooled in iced saline for 10 min. At each 500 μm interval, 30 μm sections were mounted on slides and stained with 0.2% cresyl violet solution (Sigma Chemical, St. Louis, MO) to visualize lesions. The areas of the lesions were integrated, and the results are presented as percentage of control.

### Western blot analysis

For Western blot analysis, cortical neurons and tissue samples were homogenized in a lysis buffer containing protease inhibitor 1 mM PMSF and phosphatase inhibitors 10 mM glycerophosphate, 10 mM NaF and 0.3 mM Na_3_Vo_4_. The lysates were sonicated and centrifuged, and the protein concentration was determined using a BCA protein assay kit (Jiancheng Bioengineering Institute). Equivalent amounts of protein (40 μg/lane) were loaded and separated on 10% SDS-PAGE gels, and transferred to polyvinylidene difluoride (PVDF) membranes. Membranes were blocked with 5% nonfat milk solution in Tris-buffered saline with 0.1% Triton X-100 (TBST) for 1 h, and then incubated overnight at 4˚C with the following primary antibody dilutions in TBST: anti-p-ERK1/2, ERK1/2, 1:800; p-Akt and Akt, 1:1,000 (Cell Signaling Technology, Danvers, MA). After that the membranes were washed and incubated with secondary antibody for 1 h at room temperature. The analysis software ImageJ was used to quantify the optical density of each band. The activation of Akt and ERK1/2 is presented as the ratio of phosphorylated kinase bands to the total kinase bands.

### Data analysis

Statistical analysis was performed using SPSS 16.0, a statistical software package. All data are presented as mean ± SD. Statistical evaluation of the data was performed by one-way ANOVA followed by Student-Newman-Keuls test (SNK test) for comparison of differences between the two groups by ANOVA. A value of P<0.05 was considered statistically significant. All apoptosis measures were analyzed by observers that were blinded to treatment grouping.

## Results

### CHPG attenuates neuronal damage in vitro

**Figure 1:**
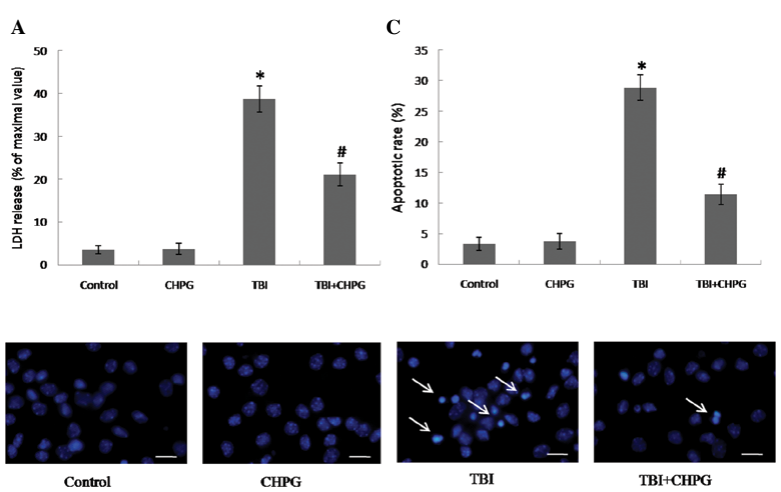
CHPG protected cultured cortical neurons against traumatic injury. CHPG at a concentration of 1 mM was added into the culture medium 30 min before traumatic injury, and the LDH release was assayed 24 h later (A). Morphology changes were observed by Hoechst 33342 staining (B). Neurons with nuclear condensation and fragmentation were counted, and the apoptotic rate is presented as a percentage of the total number of neurons (C). The arrowhead indicates karyopycnosis. Scale bars, 20 μm. The data are presented as means ± SD from six experiments. ^*^P<0.05 vs. control group; ^#^P<0.05 vs. TBI group.

To determine the potential protective effects of CHPG in an *in vitro* trauma model, LDH release and Hoechst 33342 staining were measured 24 h after mechanical injury (Fig. 1). In this model, injury to neuron cultures markedly increased LDH release, and this increase was attenuated by the addition of 1 mM CHPG. CHPG significantly reduced the neuronal apoptotic rate from 28.9±1.1% in the TBI group to 11.4±1.0% in the TBI+CHPG group (Fig. 1B and C). The results in the control group and the CHPG group were not apparently different, suggesting that CHPG in all concentrations used have no cytotoxicity.

### CHPG protects against TBI in vivo

**Figure 2:**
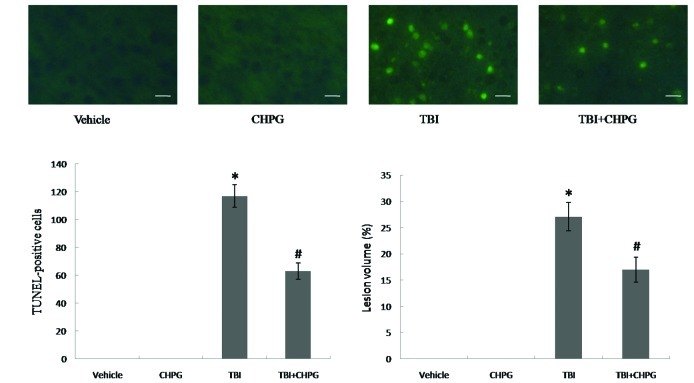
Administration of CHPG attenuated neuronal damage after TBI *in vivo*. CHPG (250 nM) was injected into right lateral ventricle in a volume of 5 μl 30 min before TBI. TUNEL staining was used to detect apoptotic cells in the cortex 24 h after TBI. Sections were examined by fluorescence microscopy (A) and the number of TUNEL-positive (apoptotic) cells was counted in 5 fields in each section (B). Lesion volume was measured by cresyl violet staining 7 days after TBI (C). Scale bars, 20 μm. The data are presented as means ± SD from seven experiments. ^*^P<0.05 vs. vehicle group; ^#^P<0.05 vs. TBI group.

To assess the efficacy of CHPG in an *in vivo* model of TBI, rats were randomly divided into the following four groups, the vehicle group (which received saline but did not undergo TBI application), the CHPG group (which was treated with CHPG but did not undergo TBI application), the TBI group (which underwent TBI application) and the TBI+CHPG group (which was treated with CHPG and underwent TBI application). Rats in the first two groups underwent similar surgical procedure, but TBI was not induced. There were no obvious TUNEL-positive cells in the vehicle group or the CHPG group (Fig. 2). However, 24 h after TBI the number of TUNEL-positive cells significantly increased (117.0±5.0 for the TBI group). Pre-treatment with 250 nM of CHPG attenuated this increase to 63±7.0 for the TBI+CHPG group. Cresyl violet staining, as a measure of cerebral lesion volume, was done 7 days after TBI, and the results demonstrated that the lesion volume of the TBI+CHPG group was significantly smaller than that of the TBI group (P<0.05).

### CHPG enhances the activation of ERK and Akt

**Figure 3:**
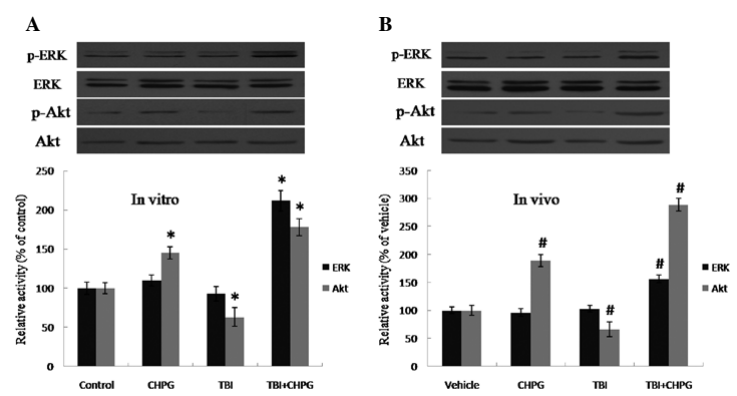
CHPG increased the activation of ERK and Akt. Western blot analysis was used to detect total as well as phosphorylated ERK and Akt *in vitro* (A) and *in vivo* (B). The data are presented as means ± SD from five experiments. ^*^P<0.05 vs. control group; ^#^P<0.05 vs. vehicle group.

To explore possible mechanisms of CHPG-induced neuroprotection, the expression levels of total and phosphorylated ERK and Akt, two pro-survival molecules downstream of mGluR5, were examined by Western blot analysis. CHPG induced an up-regulation (212±13% *in vitro* and 156±7% *in vivo*) of phosphor-ERK (p-ERK) after traumatic injury, but with no effect on control neurons or in the non-injured animals (Fig. 3). The expression of phsopho-Akt (p-Akt) was reduced following traumatic injury. CHPG treatment alone increased p-AKT levels (145±8% *in vitro* and 189±11% *in vivo*), an effect which was augmented following traumatic injury (178±9% *in vitro* and 289±10% *in vivo*).

### The inhibition of ERK and Akt partially blocked the protective effects of CHPG

**Figure 4:**
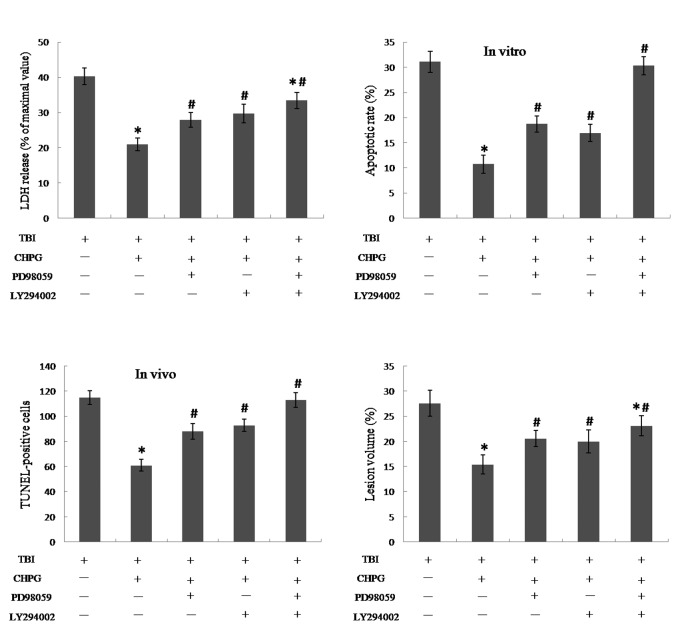
Effects of ERK and Akt inhibitors on CHPG-induced neuroprotection. CHPG was added into culture medium (1 mM) or injected into right lateral ventricle (250 nM) in the presence of the ERK inhibitor PD98059 and/or LY294002, a selective inhibitor of Akt. Then the LDH release (A), apoptosis in cultured neurons (B), TUNEL-positive cells (C) and lesion volume (D) were assayed. The data are presented as means ± SD, n=6 independent experiments (A and B), n=5 independent experiments (C and D). ^*^P<0.05 vs. TBI group; ^#^P<0.05 vs. CHPG+TBI group.

To further elucidate the mechanism of neuroprotection by CHPG, two antagonists PD98059 and LY294002 were used in both *in vitro* and *in vivo* models of TBI to block ERK and Akt activation, respectively. CHPG reduced the TBI-induced LDH release and neuronal apoptosis, and these effects were diminished by application of either PD98059 or LY294002 (Fig. 4A and B). Similar results were obtained in the *in vivo* model of TBI. Specifically, pretreatment with either PD98059 or LY294002 significantly increased the number of TUNEL-positive cells and the lesion volume compared to the TBI+CHPG group (Fig. 4C and D), suggesting that neuroprotection was partially reversed. When PD98059 and LY294002 were used together, the CHPG-induced reduction of TBI-induced apoptosis both *in vitro* and *in vivo* was abolished (Fig. 4B and C). LDH release and lesion volume were increased following co-application of PD98059 and LY294002 as compared to the TBI+CHPG group, but still lower than TBI group (P<0.05), suggesting that CHPG-induced protection was attenuated, but not totally reversed.

## Discussion

The major findings of the present study are: i) The selective mGluR5 agonist CHPG attenuated neuronal damage after traumatic injury *in vitro*; ii) CHPG reduced neuronal apoptosis and lesion volume in an *in vivo* model of TBI; iii) CHPG enhanced the expression of p-ERK and p-Akt after traumatic brain injury; iv) Activated ERK and Akt both contribute to the protective effects of CHPG against TBI.

It has long been known that group I mGluRs have a predominantly post-synaptic distribution and can mediate signal transduction through the activation of Gq-protein and phospholipase C (29). The distribution of mGluR5 is greatest in the cortex, striatum and hippocampus, all of which are sensitive to brain insults, including traumatic injury (30). The results of previous studies using general and specific antagonists of mGluR5 suggest that these receptors play important roles in central nervous system injury. For example, MPEP and the structurally-related selective mGluR5 antagonist SIB-1893 significantly attenuated post-traumatic neuronal cell death and improved functional recovery. However, the neuroprotective effects of these compounds were mediated by their antagonism of N-methyl-D-aspartate (NMDA) receptors, not by their actions on mGluR5 (31). An *in vitro* experiment showed that antisense oligodeoxynucleotides directed at mGluR1 (but not at mGluR5) was neuroprotective (18). More recently, the neuroprotective effects of mGluR5 activation were demonstrated using an *in vitro* model of β-amyloid-induced cell death (32,33). Our findings confirm their observations in *in vitro* and *in vivo* TBI models. We found that potentiating the mGluR5 with the selective agonist CHPG attenuated traumatic brain injury by inhibiting apoptosis. These findings are consistent with a previous study in CHO cells (34). Together, these data provide strong evidence for the neuroprotective role of mGluR5 in TBI and suggest for the first time that CHPG, through its activation of mGluR5, has potential therapeutic applications for TBI.

Extracellular signal-regulated protein kinase (ERK), a member of the mitogen-activated protein kinase (MAPK) family, is a potential downstream mediator of mGluR5 activity (35). ERK participates in cell survival, and numerous studies demonstrate that ERK activation by phosphorylation of both threonine and tyrosine residues is neuroprotective (23,36,37). A previous study demonstrated that administration of inhibitors of the ERK cascade reduced the recovery of cognitive and motor deficits in rats with cortical impact injury (38). As a beneficial treatment, hypothermia remarkably improved functional outcome after TBI by augmentation of ERK1/2 activation and its downstream signalling components (39). The results of the current study showed that CHPG increased ERK phosphorylation and reduced neuronal damage after TBI. Furthermore, the protective effects of CHPG were partially reversed by the selective ERK inhibitor PD98059. These findings indicate that the neuroprotective effects of CHPG are associated with an up-regulation of ERK1/2 activation.

Akt, also known as protein kinase B (PKB), is a serine/threonine kinase and plays a critical role in the modulation of cell death and survival in the adult brain (40,41). It is well-known that activation of Akt is dependent upon PI3-K, and the activation of a G protein-coupled receptor (GPCR) is required to activate PI3-K (42). As a GPCR, mGluR5 can activate the Akt pathway through PI3-K (24). In our study, the selective mGluR5 agonist CHPG significantly increased the activation of Akt and attenuated cell damage induced by TBI. Recent studies have indicated that active Akt can inactivate several pro-apoptotic target molecules such as the initiator caspase, caspase-9 (43), the proapoptotic protein Bad and the transcription factor FKHRL-1 (44,45). Another investigation shows that Akt can phosphorylate and activate the transcription factor cAMP response element (CRE)-binding protein (CREB), which is implicated in the transcription of the anti-apoptotic bcl-2 gene (46). In this study, blocking the activation of AKT by application of the selective inhibitor LY294002 partially reversed the anti-apoptotic and neuroprotective effects of CHPG. This finding suggests that the neuroprotective effects of CHPG are also mediated by Akt activation.

Cell death is divided into at least two categories, apoptosis (programmed) and necrosis (mostly non-programmed). In general, the nature of cell death is dependent on the cell types and the extent of exposure to an insult, though both forms can simultaneously occur in a tissue (47). Although we did not discriminate necrosis from apoptosis in our research, traumatic brain injury can cause neuronal damage through both forms (48), and both forms contribute to the increase of LDH release and lesion volume. Interestingly, the present work found that the anti-apoptotic effect of CHPG was abolished by inhibitors of ERK and Akt, whereas co-application of the two inhibitors did not completely reverse the CHPG-induced neuroprotective effects (as compared to that observed in the TBI group). Thus, the inhibition of CHPG's anti-apoptotic activity by co-application of ERK and Akt inhibitors does not completely reverse its neuroprotective effects. These results suggest that other mechanisms, such as anti-necrotic pathways, may also be involved in the CHPG-induced neuroprotection, a possibility which requires further studies.

In conclusion, our results provide evidence that the selective mGluR5 agonist CHPG has anti-apoptotic and neuroprotective effects in *in vitro* and *in vivo* models of TBI. The possible mechanisms through which CHPG provides neuroprotection are by activating ERK and Akt. Therefore, compounds that selectively activate mGluR5 may be promising candidates for the treatment of traumatic brain injury.

## References

[1] Langlois JA Rutland-Brown W Wald MM The epidemiology and impact of traumatic brain injury: a brief overview J Head Trauma Rehabil 21 375 378 2006 16983222 10.1097/00001199-200609000-00001

[2] Yu S Kaneko Y Bae E Severity of controlled cortical impact traumatic brain injury in rats and mice dictates degree of behavioral deficits Brain Res 1287 157 163 2009 19573519 10.1016/j.brainres.2009.06.067

[3] Mammis A McIntosh TK Maniker AH Erythropoietin as a neuroprotective agent in traumatic brain injury Review Surg Neurol 71 527 531 2009 18789503 10.1016/j.surneu.2008.02.040

[4] Finnie JW Blumbergs PC Traumatic brain injury Vet Pathol 39 679 689 2002 12450198 10.1354/vp.39-6-679

[5] McAllister TW Psychopharmacological issues in the treatment of TBI and PTSD Clin Neuropsychol 23 1338 1367 2009 19882475 10.1080/13854040903277289

[6] Narayan RK Michel ME Ansell B Clinical trials in head injury J Neurotrauma 19 503 557 2002 12042091 10.1089/089771502753754037 PMC1462953

[7] Bullock R Zauner A Woodward JJ Factors affecting excitatory amino acid release following severe human head injury J Neurosurg 89 507 518 1998 9761042 10.3171/jns.1998.89.4.0507

[8] Bullock R Zauner A Myseros JS Marmarou A Woodward JJ Young HF Evidence for prolonged release of excitatory amino acids in severe human head trauma. Relationship to clinical events Ann NY Acad Sci 765 290 298 1995 7486616 10.1111/j.1749-6632.1995.tb16586.x

[9] Faden AI Demediuk P Panter SS Vink R The role of excitatory amino acids and NMDA receptors in traumatic brain injury Science 244 798 800 1989 2567056 10.1126/science.2567056

[10] Schumann J Alexandrovich GA Biegon A Yaka R Inhibition of NR2B phosphorylation restores alterations in NMDA receptor expression and improves functional recovery following traumatic brain injury in mice J Neurotrauma 25 945 957 2008 18721106 10.1089/neu.2008.0521 PMC2946870

[11] Yurkewicz L Weaver J Bullock MR Marshall LF The effect of the selective NMDA receptor antagonist traxoprodil in the treatment of traumatic brain injury J Neurotrauma 22 1428 1443 2005 16379581 10.1089/neu.2005.22.1428

[12] Belayev L Alonso OF Liu Y Talampanel, a novel noncompetitive AMPA antagonist, is neuroprotective after traumatic brain injury in rats J Neurotrauma 18 1031 1038 2001 11686490 10.1089/08977150152693728

[13] Homayoun H Moghaddam B Group 5 metabotropic glutamate receptors: role in modulating cortical activity and relevance to cognition Eur J Pharmacol 639 33 39 2010 20371231 10.1016/j.ejphar.2009.12.042

[14] Lyeth BG Gong QZ Shields S Muizelaar JP Berman RF Group I metabotropic glutamate antagonist reduces acute neuronal degeneration and behavioral deficits after traumatic brain injury in rats Exp Neurol 169 191 199 2001 11312571 10.1006/exnr.2001.7643

[15] Gong QZ Delahunty TM Hamm RJ Lyeth BG Metabotropic glutamate antagonist, MCPG, treatment of traumatic brain injury in rats Brain Res 700 299 302 1995 8624726 10.1016/0006-8993(95)01081-6

[16] Fei Z Zhang X Bai HM Jiang XF Wang XL Metabotropic glutamate receptor antagonists and agonists: potential neuroprotectors in diffuse brain injury J Clin Neurosci 13 1023 1027 2006 17113985 10.1016/j.jocn.2005.11.042

[17] Faden AI O'Leary DM Fan L Bao W Mullins PG Movsesyan VA Selective blockade of the mGluR1 receptor reduces traumatic neuronal injury in vitro and improves outcome after brain trauma Exp Neurol 167 435 444 2001 11161632 10.1006/exnr.2000.7577

[18] Mukhin A Fan L Faden AI Activation of metabotropic glutamate receptor subtype mGluR1 contributes to post-traumatic neuronal injury J Neurosci 16 6012 6020 1996 8815884 10.1523/JNEUROSCI.16-19-06012.1996 PMC6579189

[19] Morin N Gregoire L Gomez-Mancilla B Gasparini F Di Paolo T Effect of the metabotropic glutamate receptor type 5 antagonists MPEP and MTEP in parkinsonian monkeys Neuropharmacology 58 981 986 2010 20074579 10.1016/j.neuropharm.2009.12.024

[20] Copani A Casabona G Bruno V The metabotropic glutamate receptor mGlu5 controls the onset of developmental apoptosis in cultured cerebellar neurons Eur J Neurosci 10 2173 2184 1998 9753103 10.1046/j.1460-9568.1998.00230.x

[21] Copani A Bruno V Battaglia G Activation of metabotropic glutamate receptors protects cultured neurons against apoptosis induced by beta-amyloid peptide Mol Pharmacol 47 890 897 1995 7746277

[22] Xu X Chua CC Gao J Neuroprotective effect of humanin on cerebral ischemia/reperfusion injury is mediated by a PI3K/Akt pathway Brain Res 1227 12 18 2008 18590709 10.1016/j.brainres.2008.06.018 PMC2575816

[23] Guerra B Diaz M Alonso R Marin R Plasma membrane oestrogen receptor mediates neuroprotection against beta-amyloid toxicity through activation of Raf-1/MEK/ERK cascade in septal-derived cholinergic SN56 cells J Neurochem 91 99 109 2004 15379891 10.1111/j.1471-4159.2004.02695.x

[24] Hou L Klann E Activation of the phosphoinositide 3-kinase-Akt-mammalian target of rapamycin signaling pathway is required for metabotropic glutamate receptor-dependent long-term depression J Neurosci 24 6352 6361 2004 15254091 10.1523/JNEUROSCI.0995-04.2004 PMC6729543

[25] Choe ES Wang JQ Group I metabotropic glutamate receptor activation increases phosphorylation of cAMP response element-binding protein, Elk-1, and extracellular signal-regulated kinases in rat dorsal striatum Brain Res Mol Brain Res 94 75 84 2001 11597767 10.1016/s0169-328x(01)00217-0

[26] Redmond L Kashani AH Ghosh A Calcium regulation of dendritic growth via CaM kinase IV and CREB-mediated transcription Neuron 34 999 1010 2002 12086646 10.1016/s0896-6273(02)00737-7

[27] Huang WD Fei Z Zhang X Traumatic injury induced homer-1a gene expression in cultured cortical neurons of rat Neurosci Lett 389 46 50 2005 16087291 10.1016/j.neulet.2005.07.014

[28] Fukushima M Lee SM Moro N Hovda DA Sutton RL Metabolic and histologic effects of sodium pyruvate treatment in the rat after cortical contusion injury J Neurotrauma 26 1095 1110 2009 19594384 10.1089/neu.2008.0771 PMC2848946

[29] Lujan R Nusser Z Roberts JD Shigemoto R Somogyi P Perisynaptic location of metabotropic glutamate receptors mGluR1 and mGluR5 on dendrites and dendritic spines in the rat hippocampus Eur J Neurosci 8 1488 1500 1996 8758956 10.1111/j.1460-9568.1996.tb01611.x

[30] Romano C Sesma MA McDonald CT O'Malley K Van den Pol AN Olney JW Distribution of metabotropic glutamate receptor mGluR5 immunoreactivity in rat brain J Comp Neurol 355 455 469 1995 7636025 10.1002/cne.903550310

[31] Movsesyan VA O'Leary DM Fan L mGluR5 antagonists 2-methyl-6-(phenylethynyl)-pyridine and (E)-2-methyl-6-(2-phenylethenyl)-pyridine reduce traumatic neuronal injury in vitro and in vivo by antagonizing N-methyl-D-aspartate receptors J Pharmacol Exp Ther 296 41 47 2001 11123360

[32] Movsesyan VA Stoica BA Faden AI MGLuR5 activation reduces beta-amyloid-induced cell death in primary neuronal cultures and attenuates translocation of cytochrome c and apoptosis-inducing factor J Neurochem 89 1528 1536 2004 15189356 10.1111/j.1471-4159.2004.02451.x

[33] Liu F Gong X Zhang G Marquis K Reinhart P Andree TH The inhibition of glycogen synthase kinase 3beta by a metabotropic glutamate receptor 5 mediated pathway confers neuroprotection to Abeta peptides J Neurochem 95 1363 1372 2005 16277616 10.1111/j.1471-4159.2005.03474.x

[34] Doherty AJ Palmer MJ Henley JM Collingridge GL Jane DE (RS)-2-chloro-5-hydroxyphenylglycine (CHPG) activates mGlu5, but no mGlu1, receptors expressed in CHO cells and potentiates NMDA responses in the hippocampus Neuropharmacology 36 265 267 1997 9144665 10.1016/s0028-3908(97)00001-4

[35] Yang L Mao L Tang Q Samdani S Liu Z Wang JQ A novel Ca^2+^-independent signaling pathway to extracellular signal-regulated protein kinase by coactivation of NMDA receptors and metabotropic glutamate receptor 5 in neurons J Neurosci 24 10846 10857 2004 15574735 10.1523/JNEUROSCI.2496-04.2004 PMC6730215

[36] Kuroki Y Fukushima K Kanda Y Mizuno K Watanabe Y Neuroprotection by estrogen via extracellular signal-regulated kinase against quinolinic acid-induced cell death in the rat hippocampus Eur J Neurosci 13 472 476 2001 11168553 10.1046/j.0953-816x.2000.01409.x

[37] Zhu Y Yang GY Ahlemeyer B Transforming growth factor-beta 1 increases bad phosphorylation and protects neurons against damage J Neurosci 22 3898 3909 2002 12019309 10.1523/JNEUROSCI.22-10-03898.2002 PMC6757635

[38] Dash PK Mach SA Moore AN The role of extracellular signal-regulated kinase in cognitive and motor deficits following experimental traumatic brain injury Neuroscience 114 755 767 2002 12220576 10.1016/s0306-4522(02)00277-4

[39] Atkins CM Oliva AA Jr Alonso OF Hypothermia treatment potentiates ERK1/2 activation after traumatic brain injury Eur J Neurosci 26 810 819 2007 17666079 10.1111/j.1460-9568.2007.05720.x

[40] Fukunaga K Kawano T Akt is a molecular target for signal transduction therapy in brain ischemic insult J Pharmacol Sci 92 317 327 2003 12939516 10.1254/jphs.92.317

[41] Mullonkal CJ Toledo-Pereyra LH Akt in ischemia and reperfusion J Invest Surg 20 195 203 2007 17613695 10.1080/08941930701366471

[42] Chong ZZ Li F Maiese K Activating Akt and the brain's resources to drive cellular survival and prevent inflammatory injury Histol Histopathol 20 299 315 2005 15578447 10.14670/hh-20.299 PMC2276698

[43] Cardone MH Roy N Stennicke HR Regulation of cell death protease caspase-9 by phosphorylation Science 282 1318 1321 1998 9812896 10.1126/science.282.5392.1318

[44] Datta SR Dudek H Tao X Akt phosphorylation of BAD couples survival signals to the cell-intrinsic death machinery Cell 91 231 241 1997 9346240 10.1016/s0092-8674(00)80405-5

[45] Leinninger GM Backus C Uhler MD Lentz SI Feldman EL Phosphatidylinositol 3-kinase and Akt effectors mediate insulin-like growth factor-I neuroprotection in dorsal root ganglia neurons FASEB J 18 1544 1546 2004 15319368 10.1096/fj.04-1581fje

[46] Pugazhenthi S Nesterova A Sable C Akt/protein kinase B up-regulates Bcl-2 expression through cAMP-response element-binding protein J Biol Chem 275 10761 10766 2000 10753867 10.1074/jbc.275.15.10761

[47] Majno G Joris I Apoptosis, oncosis, and necrosis. An overview of cell death Am J Pathol 146 3 15 1995 7856735 PMC1870771

[48] Sullivan PG Keller JN Bussen WL Scheff SW Cytochrome c release and caspase activation after traumatic brain injury Brain Res 949 88 96 2002 12213303 10.1016/s0006-8993(02)02968-2

